# LncRNA WWTR1-AS1 upregulates Notch3 through miR-136 to increase cancer cell stemness in cervical squamous cell carcinoma

**DOI:** 10.1186/s12905-024-02905-7

**Published:** 2024-02-08

**Authors:** Xiaofeng Zhou, Zhizun Li, Moyu Li

**Affiliations:** Department of Obstetrics and Gynecology, Bishan hospital of Chongqing medical university, Bishan Hospital of Chongqing, No. 9 Shuangxing Avenue, Biquan Street, Bishan District, 402760 Chongqing City, P. R. China

**Keywords:** WWTR1-AS1, CSCC, miR-136, Notch3, Stemness

## Abstract

**Background:**

This Study investigated the role of WWTR1-AS1 in cervical squamous cell carcinoma (CSCC).

**Results:**

WWTR1-AS1 expression was upregulated in CSCC tissues. WWTR1-AS1 was predicted to interact with miR-136, whereas correlation analysis revealed that there was no close correlation between WWTR1-AS1 and miR-136 across CSCC samples. Moreover, WWTR1-AS1 and miR-136 did not regulate the expression of each other. In addition, overexpression of WWTR1-AS1 increased the expression levels of Notch3, which could be targeted by miR-136. Cell stemness analysis indicated that the overexpression of WWTR1-AS1 and Notch3 increased CSCC cell stemness and the capacity of CSCC cell to grow as spheroids. Overexpression of miR-136 decreased CSCC cell stemness and reversed the effects of overexpression of WWTR1-AS1 on Notch3 in CSCC cells.

**Conclusion:**

Therefore, WWTR1-AS1 may upregulate Notch3 through miR-136 to increase cancer cell stemness in CSCC.

**Supplementary Information:**

The online version contains supplementary material available at 10.1186/s12905-024-02905-7.

## Introduction

Cervical cancer is among the most frequently diagnosed malignancies among females [1, 2]. Cervical cancer affected a total number of 569,847 females in 2018 [3]. The majority of cervical cancer cases are caused by HPV infections [4, 5]. Women are more frequently affected by HPV infection due to men being carriers of the virus. With increased understanding of the molecular mechanisms of HPV infection and application of HPV vaccination, the incidence and mortality rates of cervical cancer have significantly dropped during the past decade [4–7]. However, HPV vaccination does not provide benefit to individuals who are already infected. Therefore, it is crucial to have screening programs in place for early cervical cancer diagnosis [8]. Furthermore, cervical cancer can also impact HPV-negative patients [9], indicating the intricate molecular pathogenesis of cervical cancer.

The development and progression of cervical cancer entail the involvement of molecular factors [10]. Understanding of the functions of molecular pathways involved in cervical cancer accelerates the development of novel targeted therapy [11]. Long non-coding RNAs (lncRNAs) do not encode proteins. However, they regulate gene expression to modulate various aspects of cancer [12]. Therefore, regulating the expression of lncRNAs may contribute to the treatment of cancers [13]. WWTR1-AS1 is a recently identified oncogenic lncRNA in head and neck squamous cell carcinoma [14], while its role in cervical cancer is unknown. Recent studied have shown that microRNA (miR)-136 binds directly to Notch receptor 3 (Notch3) [15, 16].Our bioinformatics analysis also showed that WWTR1-AS1 might interact with miR-136, which plays tumor suppressive roles by targeting Notch3 to suppress tumor cell stemness [16]. Therefore, we hypothesized that WWTR1-AS1 upregulated Notch3 through miR-136 to increase cervical squamous cell carcinoma (CSCC) cell stemness. This study was carried out to investigate the interaction among WWTR1-AS1, miR-136 and Notch3 in CSCC, which is the major subtype of cervical cancer. Our findings could have potential advantages for the early diagnosis of CSCC.

## Materials and methods

### CSCC patients

This study enrolled a total of 60 research subjects who were diagnosed with CSCC at Bishan Hospital Affiliated to Chongqing Medical University, between May 2017 and May 2019. The age of the patients ranged from 40 to 68 years, with a mean age of 53.3 ± 6.0 years. There were 28 male and 32 female patients. The Ethics Committee of this hospital approved this study. All clinicopathological data were shown in Table 1. All 60 patients had complete medical records. All CSCC patients were diagnosed for the first time. Recurrent CSCC cases were excluded from this study. Patients complicated with other clinical disorders or with initiated therapy were also excluded. All patients were diagnosed through histopathological biopsy, during which CSCC and paired non-tumor tissue samples were collected from each patient. Tissues were stored in liquid nitrogen before use. Based on their medical record, the 60 patients included 44 HPV-positive cases (within 6 months before diagnosis) and 16 HPV-negative cases. Based on clinical stage (AJCC), there were 13, 11, 24 and 12 cases at clinical stage I, II, III and IV, respectively. All patients signed the informed consent.

**Table 1:** Clinicopathological data of 60 CSCC patients

Characteristic	Cases(*n* = 60)
Gender	
Male	28
Female	32
Age (years, 40–68)	
≤ 53	31
>53	29
HPV	
HPV positive	44
HPV negative	16
TNM stage	
I	13
II	11
III	24
IV	12

### Specimens and cells

Before therapy, fine needle aspiration was conducted to collect fresh tissues. SiHa (HPV-16 positive) human CSCC cell line (ATCC, USA) was used. SiHa cells were cultivated in EMEM. The cell culture was maintained at 95% humidity, 37 °C and 5% CO_2_. Cells were harvested when about 85% confluence was reached to perform subsequent experiments.

### Transient transfections

WWTR1-AS1 and Notch3 expression vectors were prepared using pcDNA3.1 as the backbone vector. Negative control (miR-NC mimics, Sigma-Aldrich), miR-136 mimic and miR-134-inhibitor were purchased from Sigma-Aldrich (USA). The sequences for miR-NC, miR-136 mimics, miR-136 inhibitors and inhibitor NC were: 5’-GUCCCAC-UUCGACCGUGCUUCCA-3’, 5’-ACUCCAUUUGUUUUGAUGAUGGA-3’, 5’-CAUCAUCGUCUCAAAUGAGUCU-3’, and 5’-UGUGCCUAUGUGAUGAUGUAC-3’, respectively. SiHa cells at 50–70% confluence were transfected with 10 nM expression vector and (co-transfection)/or 40 nM miRNA using lipofectamine 2000 (Invitrogen) following the instruction of manufacturers. Untransfected cells were used as the Control (C) cells. NC cells were miR-NC mimics - or empty vector-transfected cells. Subsequent experiments were performed 48 h later.

### Dual luciferase activity assay

Dual-luciferase reporter assay was performed as previously described [17]. The WWTR1-AS1 wild-type (WT) and mutant (MUT) luciferase system were constructed using pGL3 as the vector backbone. Lipofectamine 2000 (Invitrogen) was used to co-transfect WWTR1-AS1 (WT/MUT) + miR-136 mimic (miR-136 group) or WWTR1-AS1 (WT/MUT) + NC miRNA (C group) into SiHa cells. Luciferase activity was measured and compared 48 h later using a Dual-luciferase Reporter Assay System (Promega).

### RNA preparation

RNA was extracted from paired tissue samples and in vitro-cultured cells, followed by quantification using a NanoDrop spectrophotometer (NanoDrop Technologies). Subsequently, DNA removal was carried out using gDNA eraser (Takara Bio).

### RT-qPCR

Total RNA reverse transcriptions and qPCR reactions were conducted using BlazeTaq™ One-Step SYBR Green RT-qPCR Kit (Genecopoeia) to detect the expression of WWTR1-AS1, miR-136 AND Notch3 mRNA with GAPDH as the endogenous control. Primer used were as follows: GAPDH forward: 5’-GGTGAAGGTCGGAGTCAACG-3’, reverse: 5’-CAAAG TTGTCATGGATGHACC-3’; WWTR1 AS1 forward: 5’-GATGCCTCCTCGCCAGACCA-3’, reverse: 5’-TACTTAGTGGCT- CAGGTCTC-3’; Notch3 forward: 5’-GTCTTCCAGATTCTCATCC-3’, reverse: 5’-ATCCACAGCATTGACATC-3’; miRNA-136 forward: 5’-ACUCCAUUUGUUUUGAUGAUGGA-3’, reverse: 5’-UCCAUCAUCAAAACAAAUGGAGU-3’; U6 forward: 5’-GCTTCGGCAGCACATATACTAAAA-3’, reverse: 5’-CGCTTCACGAATTTGCGTGTCA-3’.

### Western blot analysis

In vitro cultivated cells were subjected to total protein isolation using RIPA buffer (Invitrogen), and the protein concentration was quantified using a BCA assay (Invitrogen). Following denaturation at 95 °C for 10 min, SDS-PAGE gel (10%) was used to separate proteins, and separated proteins were transferred to PVDF membranes. Blocking was performed in PBS containing 5% non-fat milk, followed by incubation with rabbit primary antibodies of Notch3 (1:1,000, ab23426, Abcam) and GAPDH (1: 1,000, ab8245, Abcam) at 4 °C for 15 h. After that, secondary antibody incubation was performed. Signals were normalized using Image J V 1.6 software.

### Cell stemness assay

SiHa cells collected at 48 h post-transfection and washed with cold PBS, followed by incubation with immunoglobulin (Ig) G1-PE (Miltenyi Biotec) or phycoerythrin (PE)-conjugated CD133, CD44 or CK17 (Biosciences) at 4 °C for 25 min. Following that, CD133 + cells were separated by performing flow cytometry.

### Statistical analysis

Data from 3 independent replicates were presented as the mean ± standard deviation (SD). Paired t-tests were employed to compare paired tissue samples, while unpaired t-tests were utilized for comparing two independent groups. For the comparison of multiple groups, a one-way ANOVA and Tukey’s test were employed. Linear regression and Pearson’s analysis were used to analyze correlations. Kaplan-Meier curves were generated using SPSS, and P-values were calculated using the log-rank test. *P* < 0.05 was considered as statistically significant.

## Results

### Expression of WWTR1-AS1 and miR-136 in CSCC

**Fig. 1:**
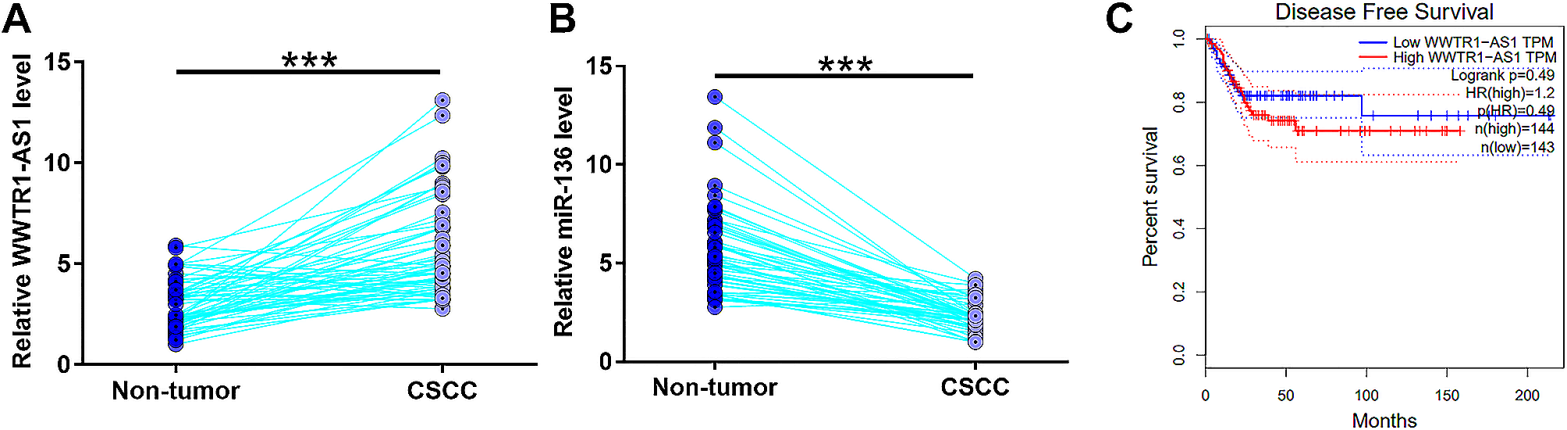
The expression of WWTR1-AS1 and miR-136 was altered in CSCC. The differential expression of WWTR1-AS1 (**A**) and miR-136 (**B**) was analyzed in paired CSCC and non-tumor tissues collected from the 60 CSCC patients. ***, *p* < 0.001. Survival data (disease-free) in TCGA dataset were explored using GEPIA (http://gepia.cancer-pku.cn/) to analyze the correlation between WWTR1-AS1 expression and the survival of CSCC patients. Kaplan-Meier curve were used by SPSS. *p* < 0.05 was considered as statistically significant (**C**)

The expression of WWTR1-AS1 and miR-136 was evaluated in paired CSCC and non-tumor tissues collected from the 60 CSCC patients including 28 males and 32 females. There were 44 HPV-positive cases and 16 HPV-negative cases. Based on clinical stage (AJCC), there were 13, 11, 24 and 12 cases as clinical stage I-IV, respectively (Table 1). Compared with non-tumor tissues, WWTR1-AS1 was highly expressed in CSCC tissues (Fig. 1A, *p* < 0.001), while the expression leves of miR-136 were significantly decreased in CSCC tissues (Fig. 1B, *p* < 0.001). HPV infections showed no effect on the expression of WWTR1-AS1 (Fig. S1). The survival data (disease-free) in TCGA dataset using GEPIA (http://gepia.cancer-pku.cn/) revealed that the expression levels of WWTR1-AS1 were slightly correlated with the poor disease-free survival rate of CECC patients (HR = 1.2, *p* = 0.46, Fig. 1C).

### WWTR1-AS1 was not correlated with miR-136

**Fig. 2:**
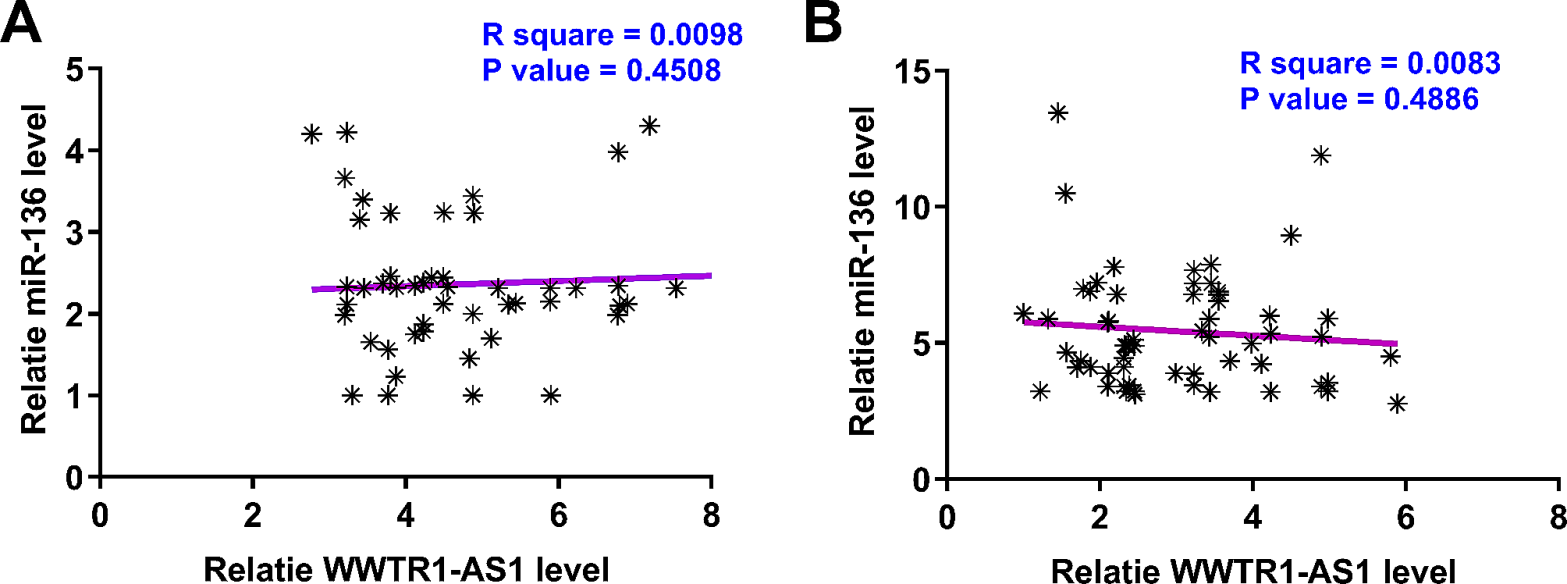
WWTR1-AS1 and miR-136 were not significantly correlated with each other. Pearson’s correlation coefficient analysis was performed to analyze the correlation between the expression levels of WWTR1-AS1 and miR-136 across both CSCC (**A**) and non-tumor (**B**) tissues

Correlations between WWTR1-AS1 and miR-136 across both CSCC (Fig. 2A) and non-tumor (Fig. 2B) tissues were analyzed. It was observed that the expression of WWTR1-AS1 and miR-136 was not correlated with each other across two types of samples from the 60 CSCC patients.

### WWTR1-AS1 and miR-136 did not regulate the expression of each other

**Fig. 3:**
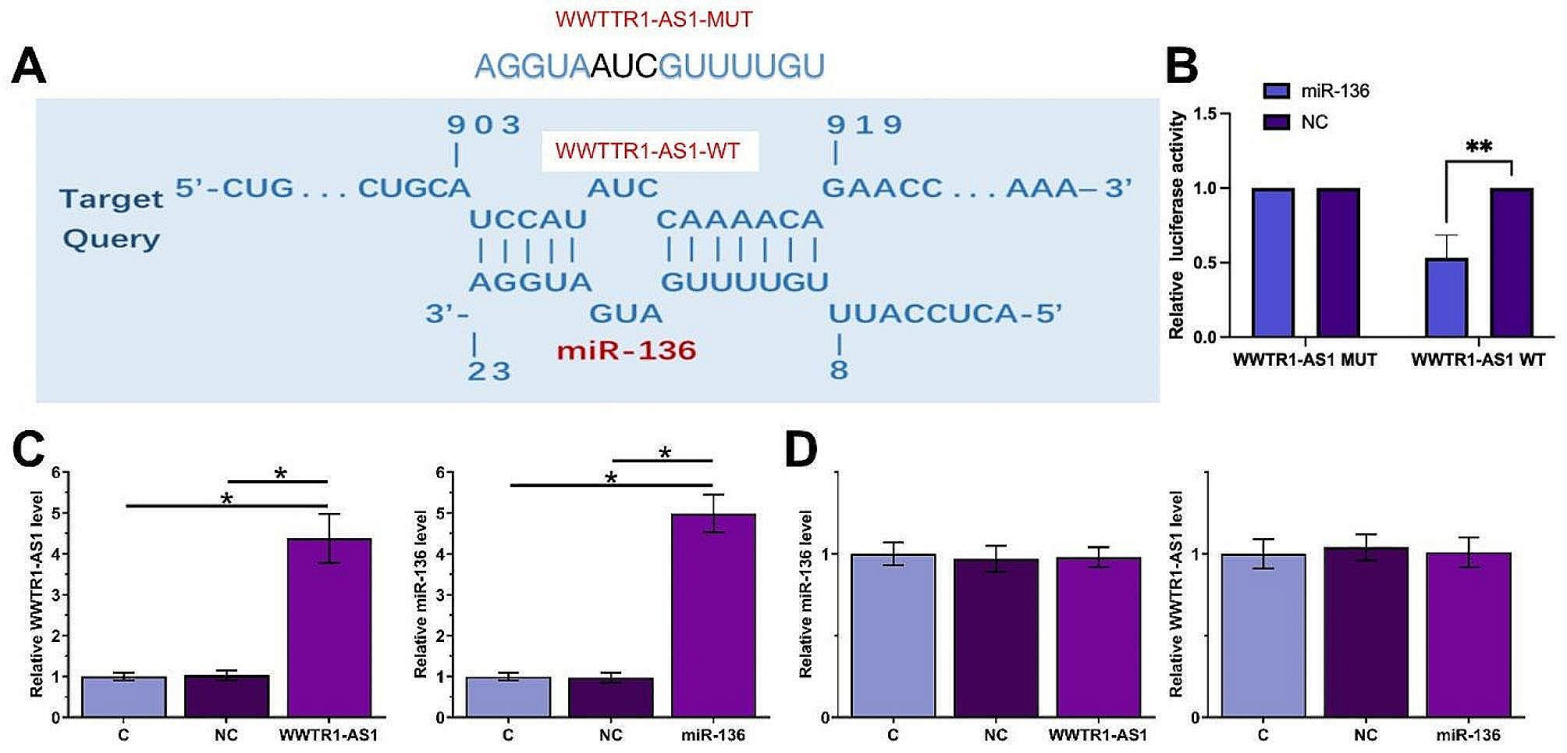
WWTR1-AS1 and miR-136 interacted with each other but did not regulate the expression of each other. IntaRNA 2.0 was applied to predict the binding of WWTR1-AS1 to miR-136 (**A**), which was further confirmed by dual luciferase activity assay (**B**). SiHa cells were transfected with either WWTR1-AS1 expression vector or miR-136 mimic, and the overexpression of WWTR1-AS1 WT/MUT and miR-136 was confirmed by RT-qPCR (**C**). The role of WWTR1-AS1 WT/MUT and miR-136 in regulating the expression of each other was studied by performing RT-qPCR (**D**). Control (**C**) cells were untransfected cells. NC cells were miR-NC mimics - or empty vector-transfected cells. *, *p* < 0.05

The interaction between WWTR1-AS1 and miR-136 was assessed using IntaRNA 2.0. It was observed that WWTR1-AS1 and miR-136 might form multiple base pairings. (Fig. 3A). Dual luciferase activity assay showed that miR-136 transfection reduced the relative luciferase activity (Fig. 3B, *p* < 0.05), indicating that there was a direct interaction between WWTR1-AS1 and miR-136. SiHa cells were transfected with either WWTR1-AS1expression vector or miR-136 mimic, and the overexpression of WWTR1-AS1 and miR-136 was confirmed by RT-qPCR (Fig. 3C, *p* < 0.05). Compared with the C and NC groups, overexpression of WWTR1-AS1 and miR-136 did not affect the expression of each other (Fig. 3D).

### Overexpression of WWTR1-AS1 led to the upregulation of Notch3

**Fig. 4:**
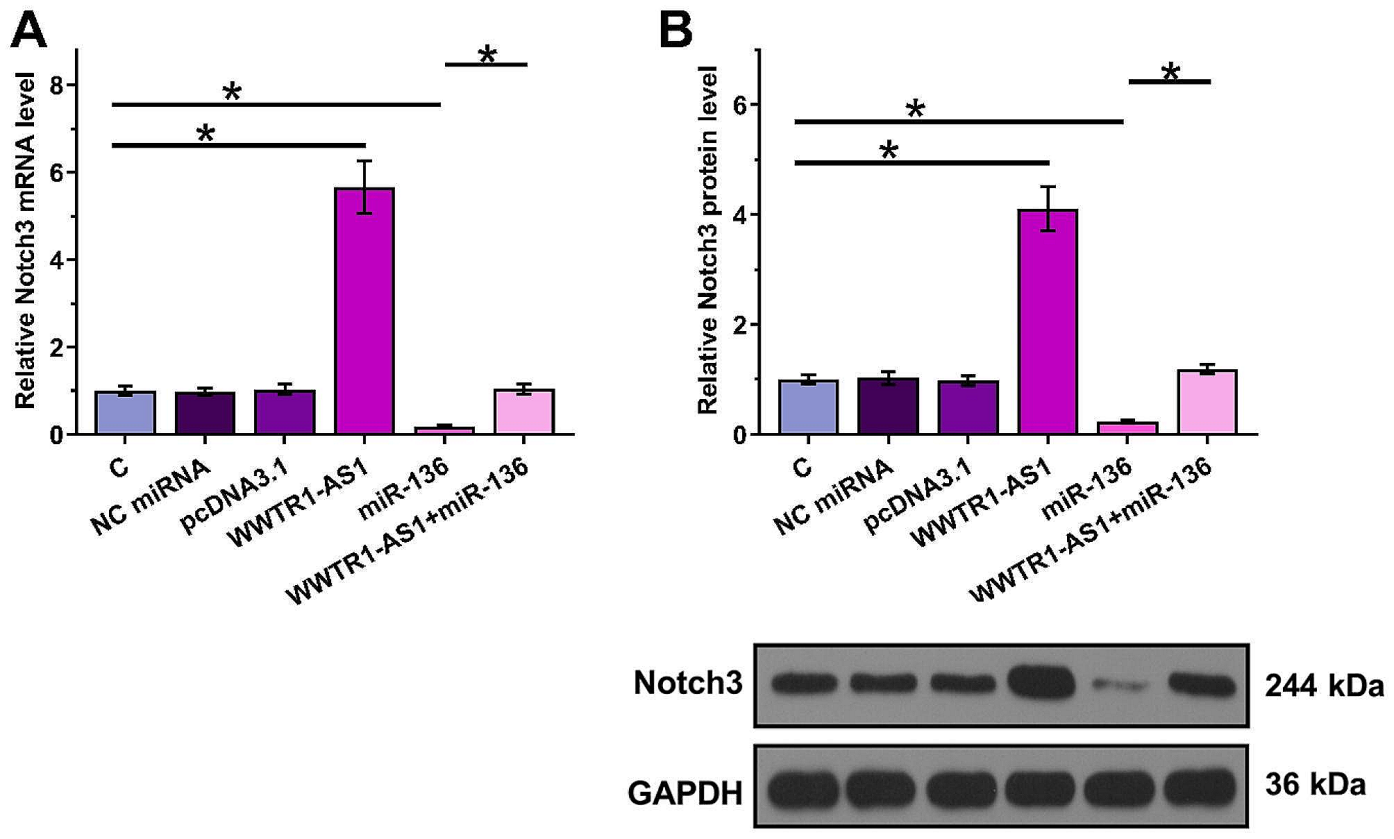
The overexpression of WWTR1-AS1 upregulated the expression of Notch3 To test whether WWTR1-AS1 can sponge miR-136, the effects of WWTR1-AS1 and miR-136 overexpression on the expression nof Notch3, a target of miR-136, were analyzed by RT-qPCR (**A**) and Western blot analysis (**B**). Control (**C**) cells were untransfected cells. NC cells were miR-NC mimics - or empty vector-transfected cells. *, *p* < 0.05

To test whether WWTR1-AS1 can sponge miR-136, the effects of WWTR1-AS1 overexpression, miR-136 overexpression and miR-136 inhibition on Notch3, a target of miR-136, were analyzed by RT-qPCR (Fig. 4A and Fig. S2A) and Western blot analysis (Fig. 4B and Fig. S2B). Compared with the C group, overexpression of miR-136 decreased the expression levels of Notch3, while inhibition of miR-136 increased the expression levels of Notch3 (Fig. 4C and Fig. S4) (*p* < 0.05). In contrast, overexpression of WWTR1-AS1 played an opposite role and reduced the effects of overexpression of miR-136 on the expression of Notch3 (*p* < 0.05).

### WWTR1-AS1 increased SiHa cell stemness by regulating the miR-136/Notch3 axis

**Fig. 5:**
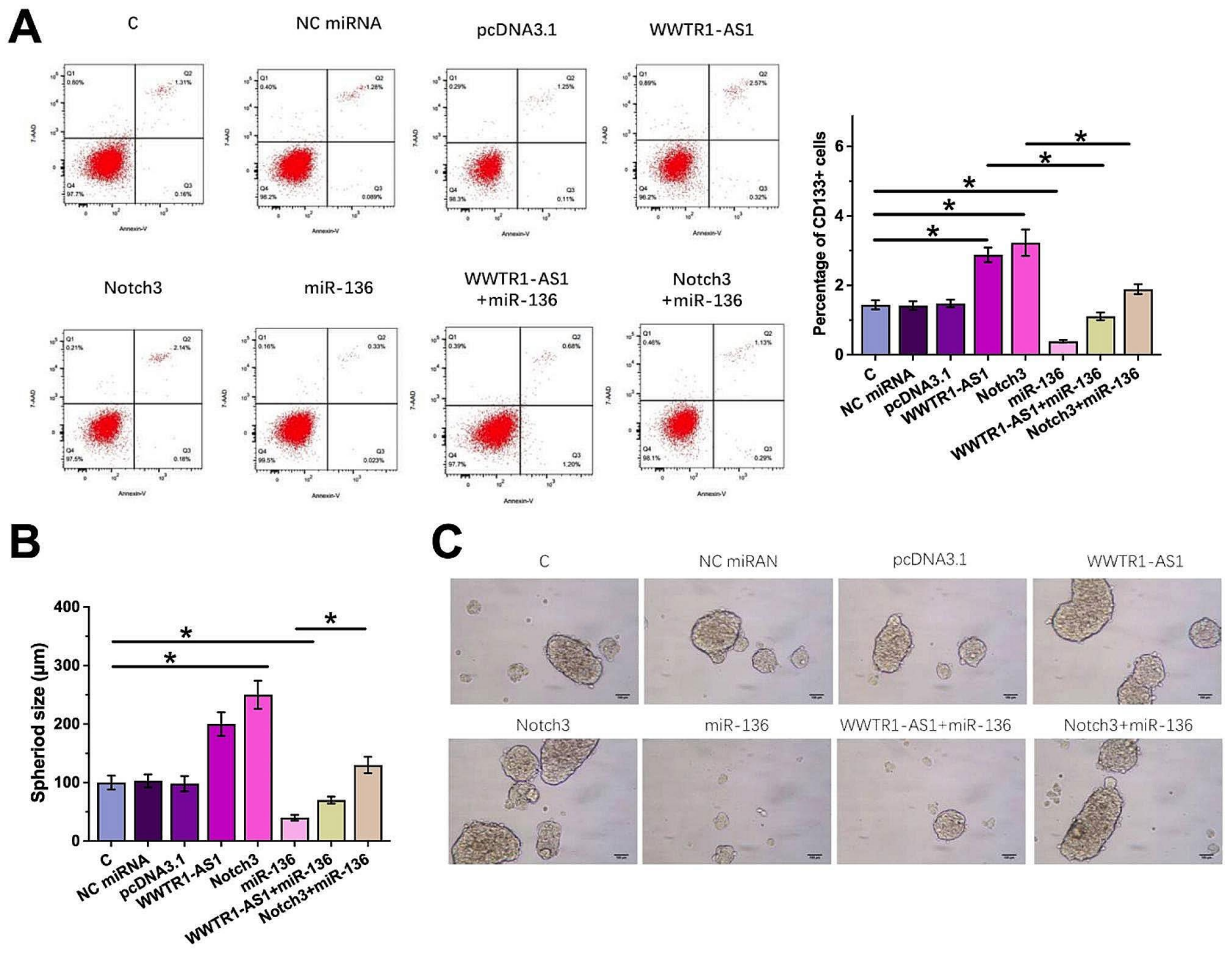
WWTR1-AS1 increased SiHa cell stemness by regulating the miR-136/Notch3 axis. The effects of WWTR1-AS1, miR-136 and Notch3 overexpression on the stemness of SiHa cells were analyzed by performing stemness assay. Control (**C**) cells were untransfected cells. NC cells were miR-NC mimics - or empty vector-transfected cells. *, *p* < 0.05

The effects of the overexpression of WWTR1-AS1, miR-136 and Notch3 on the stemness of SiHa cells were assessed by performing stemness assay (Fig. 5A and Fig. S3A). Compared with the control group (C group), overexpression of WWTR1-AS1 and Notch3 was observed to enhance the stemness of CSCC cells, reflected by the increase in the percentage of CD133 + cells [18, 19]. The overexpression of miR-136 led to a reduction in CSCC cell stemness and counteracted the effects of WWTR1-AS1 and Notch3 overexpression on CSCC cell stemness. It was worth noting that CD44 or CK17, which are markers of stemness, exhibited similar results. In addition, one of the key characteristics of stemness is its role in promoting the growth of cells in the form of spheroids [20]. We demonstrated that the overexpression of WWTR1-AS1 and Notch3 increased the ability of CSCC cells to form spheroids. Conversely, the overexpression of miR-136 decreased this spheroid-forming capacity and countered the effects of WWTR1-AS1 and Notch3 overexpression. However, the inhibition of miR-136 resulted in an increased spheroid-forming capacity of CSCC cells (Fig. 5B and C and Fig. S3B and C). Taken together, these results suggested that WWTR1-AS1 increased SiHa cell stemness by regulating the miR-136/Notch3 axis.

## Discussion

CSCC treatments traditionally include surgery, chemotherapy, and radiation. Despite these interventions, overall survival rates have not seen substantial improvements, highlighting the need for novel approaches. Immunotherapy, which has demonstrated effectiveness in various cancer types, has emerged as a promising avenue. Recent developments in immunotherapy have expanded the possibilities, including the potential for therapeutic vaccines and other tailored treatment strategies in cervical cancers. These advancements contribute to the pursuit of more precise and effective therapies [21]. The molecular mechanisms unveiled in this study provide insights into the enhancement of CSCC screening and potentially refining treatment approaches.

The present study investigated the interplay among WWTR1-AS1, miR-136 and Notch3 in CSCC. We observed significant alterations in the expressions of WWTR1-AS1 and miR-136 in CSCC. Notably, WWTR1-AS1 was identified as a sponge for miR-136, leading to the upregulation of Notch3 and consequently enhancing cell stemness. The function of WWTR1-AS1 has only been investigated in head-neck squamous cell carcinoma, in which WWTR1-AS1 is upregulated and promotes cancer cell proliferation, invasion and migration [14]. We observed the upregulation of WWTR1-AS1 in CSCC, and overexpression of WWTR1-AS1 increased cancer cell stemness. Therefore, WWTR1-AS1 is likely an oncogenic lncRNA in CSCC. It is known that HPV infections may promote the development of CSCC by regulating the expression of lncRNAs [22]. In this study, we observed no significant differences in the expression levels of WWTR1-AS1 in CSCC tissues between HPV-positive and HPV-negative patients. This suggests that WWTR1-AS1 may be involved in CSCC through an HPV-independent mechanism.

MiR-136 suppresses several types of cancers including CSCC [23, 24]. It has been reported that miR-136 is downregulated in cervical cancer, suppresses tumor cell apoptosis and induces cell apoptosis by targeting E2F1 [24]. In a recent study, miR-136 was reported to suppress cell stemness [16]. Additionally, Zong et al. [15]. demonstrated that miR-136 could target the NOTCH3 pathway. However, there is no there is no prior study investigating the specific mechanism of the miR-136/Notch3 interaction in cervical squamous cell carcinoma in cervical squamous cell carcinoma.

Interestingly, in this study, we showed that WWTR1-AS1 and miR-136 could interact with each other. However, there is no significant correlation between them in both CSCC and non-tumor tissues. Furthermore, WWTR1-AS1 and miR-136 do not mutually regulate each other’s expression. A similar lack of regulatory influence between SNHG10 and miR-543 has been documented [25]. In our study, we similarly observed an interaction between WWTR1-AS1 and miR-136, yet neither of them exhibited regulatory control over the expression of the other, and they did not display a significant correlation. Therefore, WWTR1-AS1 was unlikely a target of miR-136. However, WWTR1-AS1 reduced the effects of miR-136 on the expression of Notch3 and cancer cell stemness. Furthermore, considering that WWTR1-AS1 directly binds to miR-136 and miR-136 directly targets Notch [15, 16], our hypothesis was that WWTR1-AS1 promotes Notch3 expression by acting as a miR-136 sponge. Indeed, we observed an upregulation of WWTR1-AS1 expression in CSCC tissues and predicted its interaction with miR-136. While the correlation analysis indicated no significant correlation between WWTR1-AS1 and miR-136, we observed that the overexpression of WWTR1-AS1 increased the expression levels of Notch3, a target of miR-136. Our study demonstrated that miR-136 could also target Notch3 to decrease cancer cell stemness in CSCC. In summary, although we have provided initial insights into the relationship between WWTR1-AS1 and miR-136 and have shown that the lncRNA WWTR1-AS1 upregulates Notch3 through miR-136 to enhance cancer cell stemness in CSCC, further evidence is required to hold the reliability of our findings. For instance, additional analysis involving stemness markers such as Nanog, OCT-4, and SOX-2 is needed to further elucidate the impact of WWTR1-AS1 on cancer cell stemness.

In conclusion, WWTR1-AS1 was upregulated, and miR-136 was downregulated in CSCC. WWTR1-AS1 may sponge miR-136 to upregulate Notch3, thereby increasing cell stemness.

### Electronic supplementary material

Below is the link to the electronic supplementary material.

Supplementary Material 1

Supplementary Material 2

Supplementary Material 3

## Data Availability

The datasets generated and/or analysed during the current study are available in the JIANGUOYUN repository, https://www.jianguoyun.com/p/DdTgaZcQ3u3kChi_zagFIAA. Also, the datasets could be requested from the corresponding author.
