# Supplementary material for: LncRNA WWTR1-AS1 upregulates Notch3 through miR-136 to increase cancer cell stemness in cervical squamous cell carcinoma
Source: BMC Womens Health. 2024 Feb 8;24:104. doi: 10.1186/s12905-024-02905-7 (PMC10851613; doi:10.1186/s12905-024-02905-7)

Supplemental 1 Relative WWTR1-AS1 level in HPV-negative or HPV-positive patients.


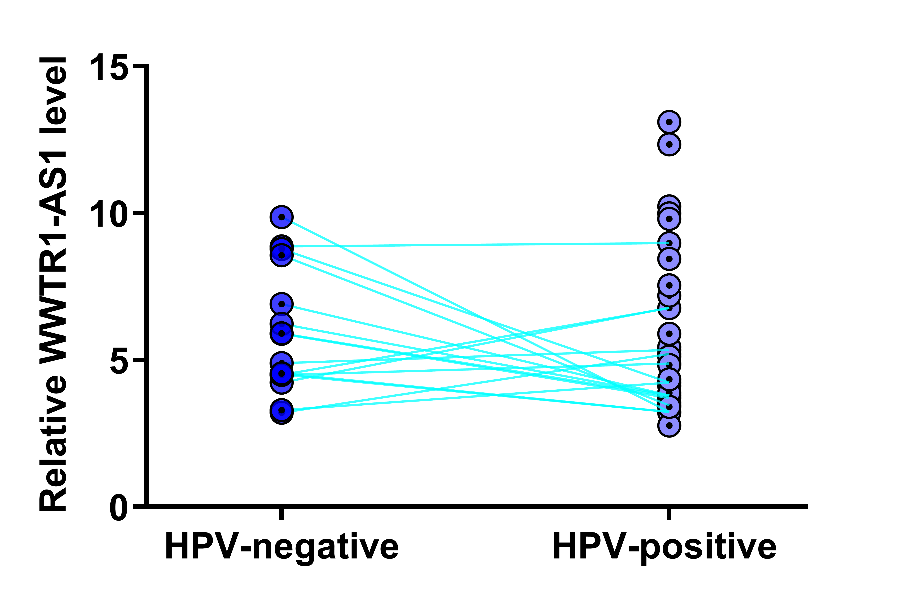


Supplemental 2 miR-136 inhibition led to the upregulation of Notch3

To test whether WWTR1-AS1 can sponge miR-136, the effects of WWTR1-AS1 and miR-136 overexpression on Notch3, a target of miR-136, were analyzed by RT-qPCR (A) and western blot (B). Control (C) cells were untransfected cells. NC cells were miR-NC mimics - or empty vector-transfected cells. *,p<0.05


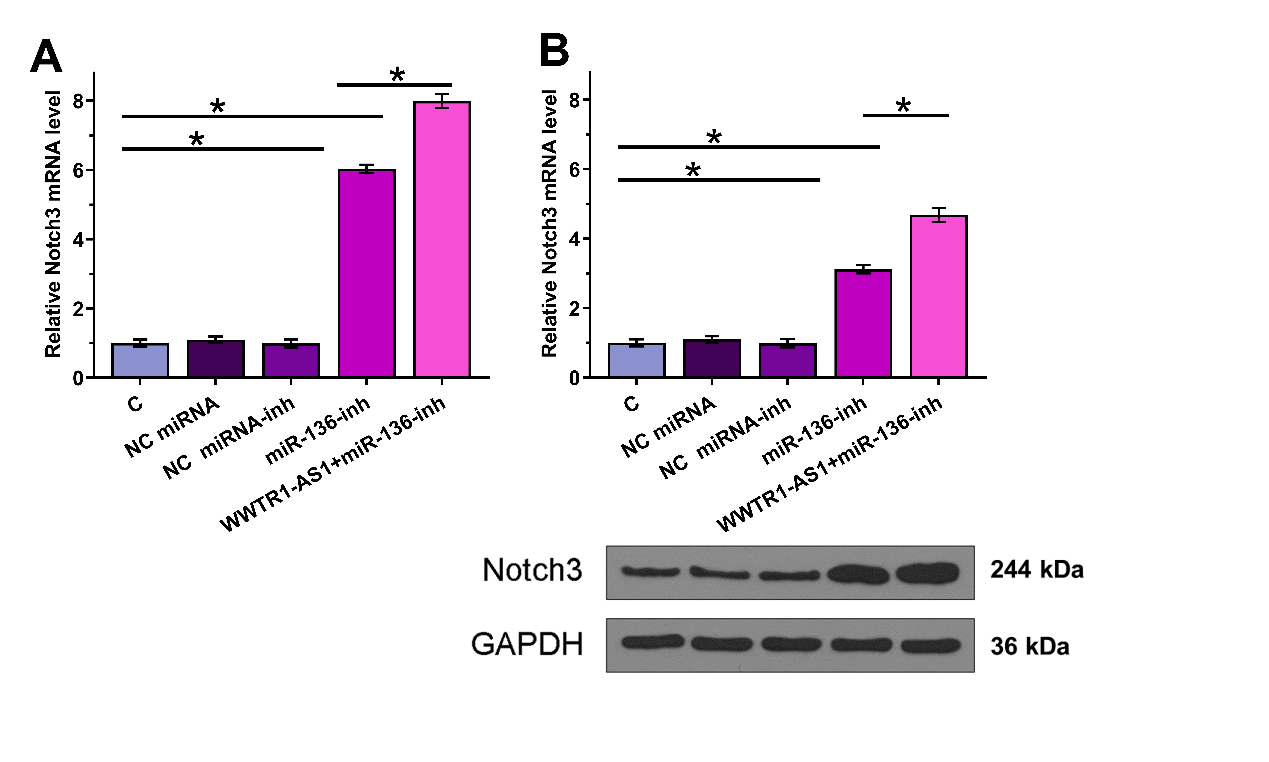


Supplemental 3 WWTR1-AS1 increased the stemness of SiHa cells by regulating miR-136/Notch3 axis

The effects of WWTR1-AS1, Notch3 overexpressio n and miR-136 inhibition on the stemness of SiHa cells were analyzed by performing stemness assay. Control (C) cells were untransfected cells. NC cells were miR-NC mimics - or empty vector-transfected cells. *,p<0.05


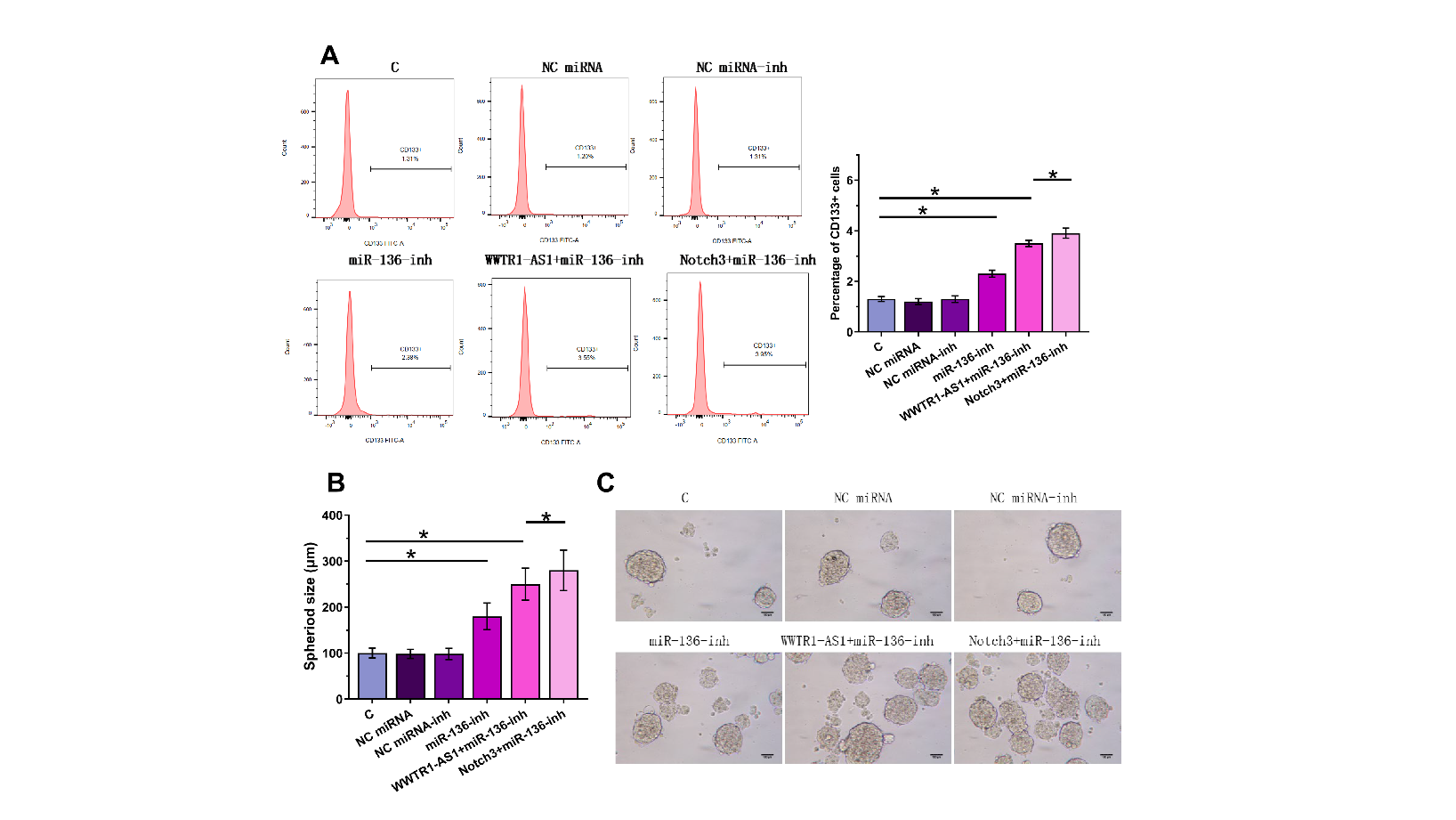


Supplemental 4 The level of Notch3 successfully overexpressed in SiHa cells were analyzed by RT-qPCR (A and B). Relative miR-136 level when inhibited was analyzed by RT-qPCR (C). C (Control) cells were untransfected cells. NC (negative control) cells were miR-NC mimics - or empty vector-transfected cells, inh-NC (miRNA inhibitor negative control) cells were miRNA inhibitor-NC-transfected cells. *,p<0.05


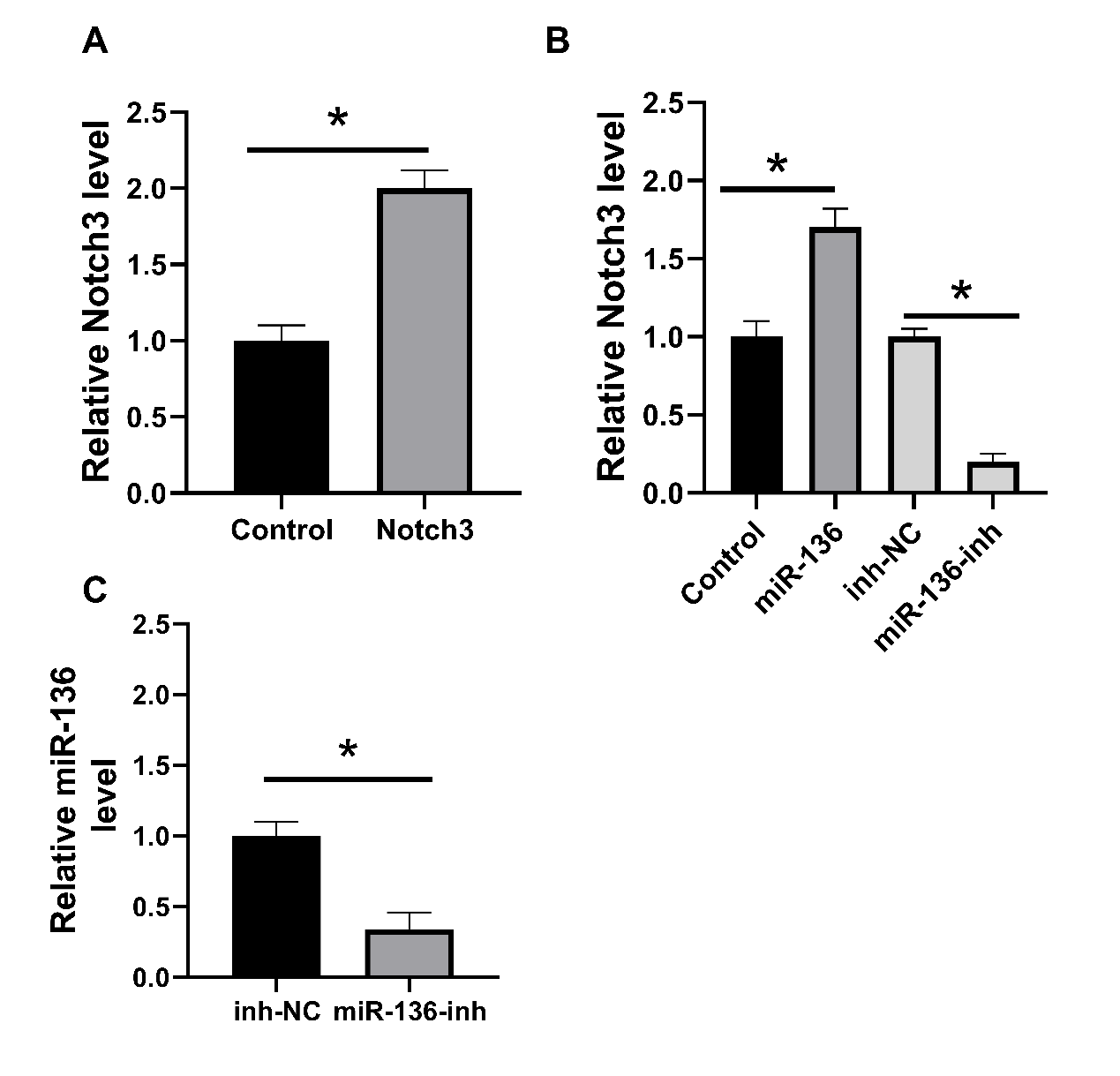

Supplement: Supplementary file 1 — Supplementary Material 1 [file 12905_2024_2905_MOESM1_ESM.docx]
