# Supplementary figures and images for: LncRNA WWTR1-AS1 upregulates Notch3 through miR-136 to increase cancer cell stemness in cervical squamous cell carcinoma
Source: BMC Womens Health. 2024 Feb 8;24:104. doi: 10.1186/s12905-024-02905-7 (PMC10851613; doi:10.1186/s12905-024-02905-7)

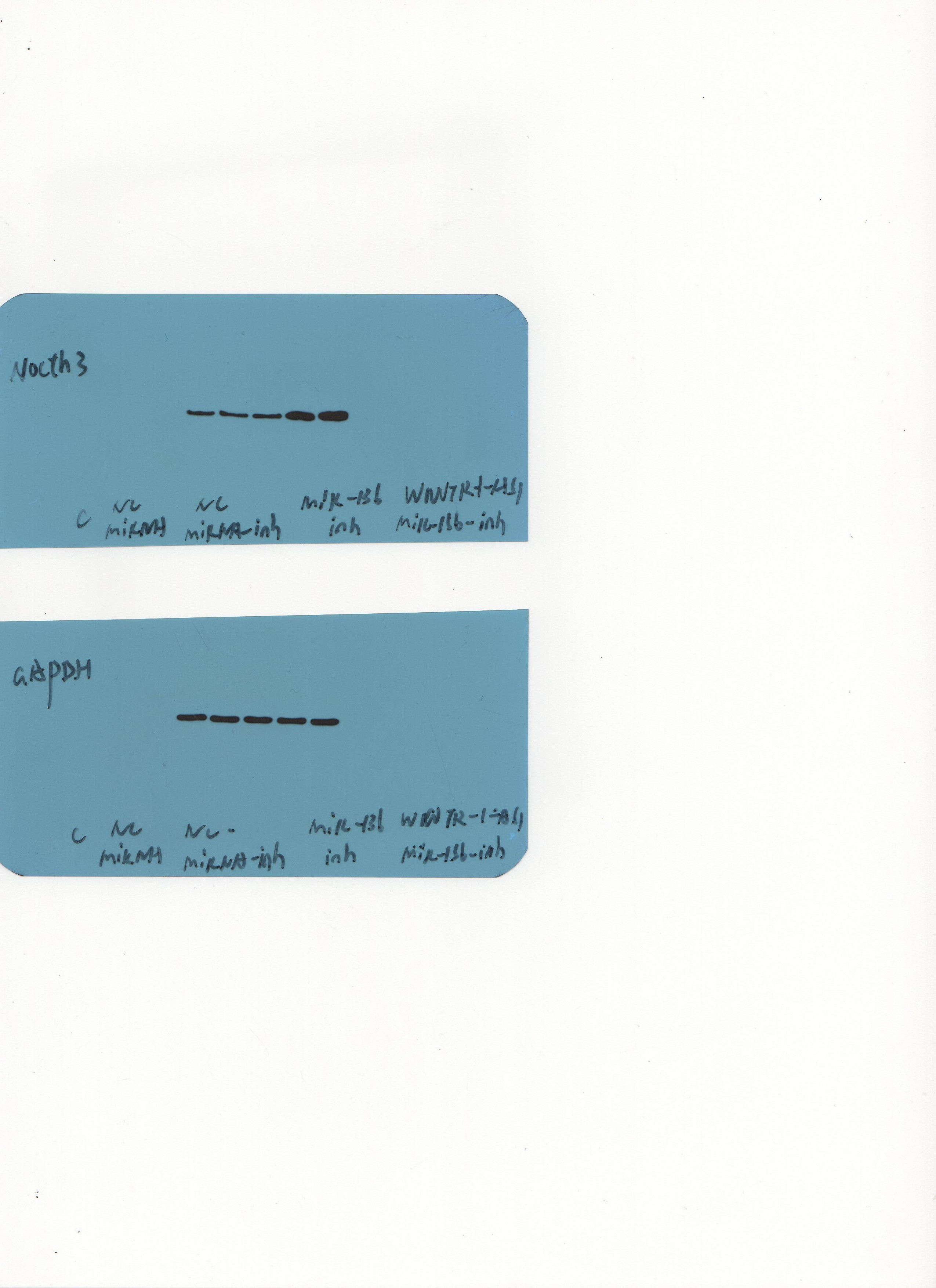

Supplement: Supplementary file 2 — Supplementary Material 2 [file 12905_2024_2905_MOESM2_ESM.png]

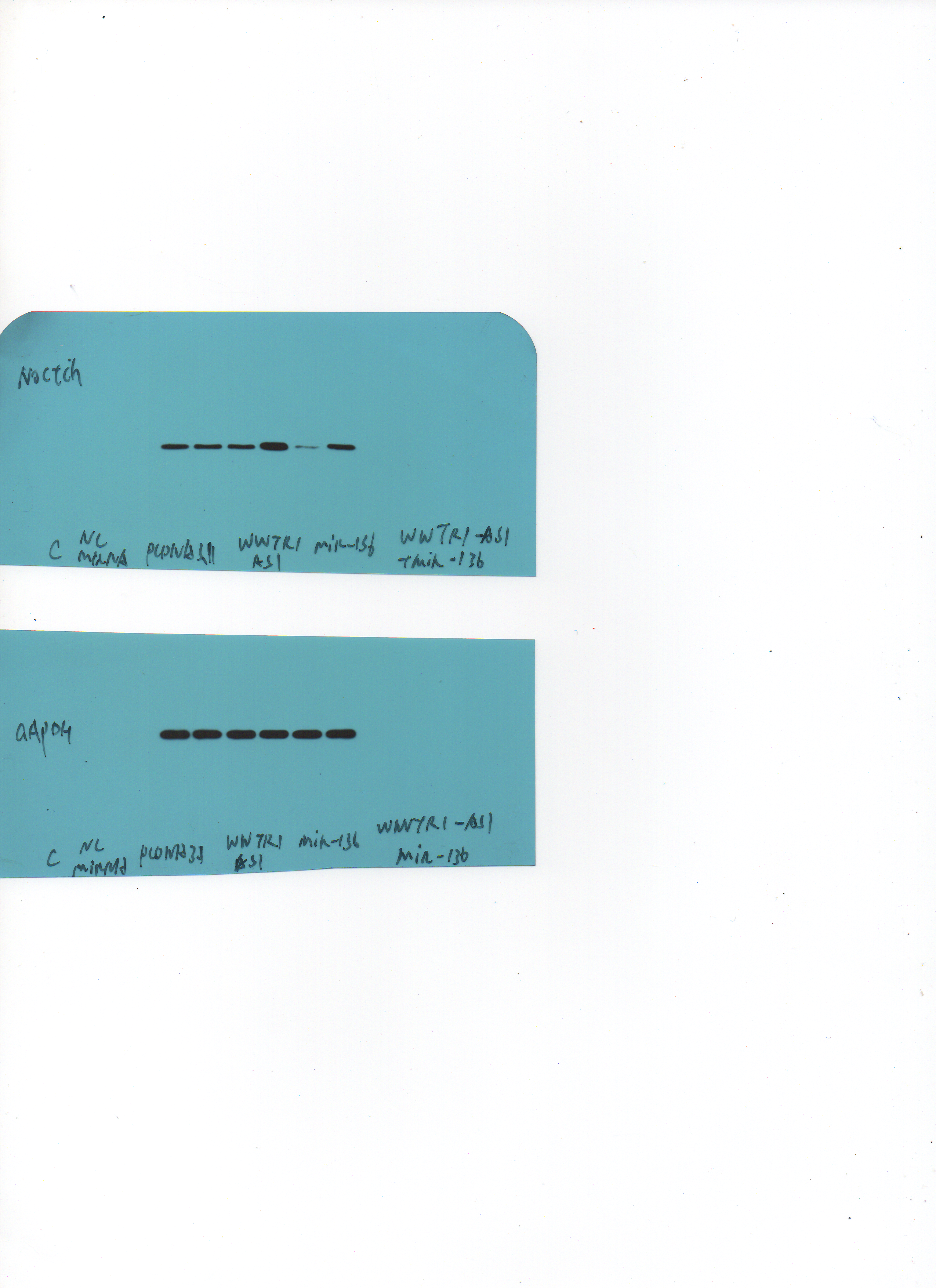

Supplement: Supplementary file 3 — Supplementary Material 3 [file 12905_2024_2905_MOESM3_ESM.png]
